# Inverse association between adult height and diabetes risk in a cohort study of Chinese population

**DOI:** 10.1038/s41598-023-47474-1

**Published:** 2023-11-27

**Authors:** Xiaoli Li, Tiantian Cheng, Lina Leng, Guangyao Song, Huijuan Ma

**Affiliations:** 1 Department of Rheumatology, Xingtai People’s Hospital, Xingtai, 054000 China; 2 Department of Internal Medicine, Hebei Medical University, Shijiazhuang, 050017 China; 3 Department of Endocrinology, The First Hospital of Hebei Medical University, Shijiazhuang, 050000 China

**Keywords:** Endocrinology, Health care, Risk factors

## Abstract

Recent studies linking adult height to diabetes risk remain controversial and few were from Asia. This study, therefore, aimed to explore the association of adult height with diabetes risk in a Chinese population. This retrospective cohort study was a secondary analysis of data from the DATADRYAD website, involving 211,172 non-diabetic individuals aged ≥ 20 years from the health screening program in China. Cox regression models were employed to evaluate hazard ratios (HRs) with 95% confidence interval (CI) of diabetes related to height. During an average 3.12-year follow-up, 4156 (1.97%) subjects reported developing diabetes. After adjusting for potential confounding factors, an inverse association of height with diabetes risk was observed among men and women [HR per 10 cm (95% CI), 0.78 (0.73–0.83) and 0.76 (0.68–0.86), respectively]. Moreover, subgroup analyses indicated the inverse association was only detected in individuals with aged < 70 years, fasting plasma glucose (FPG) < 6.1 mmol/L, and men with body mass index (BMI) < 28 kg/m^2^. In brief, height is inversely associated with diabetes risk in Chinese adults. Specifically, this association appears to be more pronounced in individuals with aged < 70 years, FPG < 6.1 mmol/L, and men with BMI < 28 kg/m^2^.

## Introduction

The dramatic rise in the prevalence of diabetes has become an increasingly serious problem in developing countries^1^. In China, with rapid economic development and urbanization, these risk factors, such as nutrition transitions, obesity, physical inactivity and aging, have become the main cause for persistent high growth in the prevalence of diabetes^2^. Additionally, increasing evidence has reported nutritional status during pregnancy or childhood is correlated with increased risk of diabetes and other chronic diseases^3–6^, and “thrifty phenotype hypothesis” may be one of the important mediating mechanisms^4^, which associates the adverse environment in early life with the risk of chronic diseases in later life ^6–8^.

Adult height, a comprehensive indicator of children's growth environment, reflects the nutritional status of children to a certain extent^9^. Persistent growth retardation from pregnancy to two years old is strongly correlated with shorter stature in adulthood^3,10^. Since 1991, a significant correlation between short stature and glucose intolerance in adults was first reported^11^. Subsequently, several studies reported adult height was inversely related to diabetes in white Americans^12^, Europeans^13,14^ and South Koreans^15^, whereas in Portuguese^16^ and African Americans^12^, no significant association was observed. In a recent cohort study in Namibia^17^ and a meta-analysis^18^, taller height was associated with decreased risk of diabetes only in women rather than men. Overall, the limited evidence for the association of adult height with diabetes risk remains controversial, and little evidence was from Asia. Hence, the purpose of this study, involving 211,172 non-diabetic individuals aged ≥ 20 years from the health screening program, was to assess the association of adult height with diabetes risk in the Chinese population.

Notably, in the initial study^19^, the authors explored the relationship between BMI and diabetes risk, and uploaded the relevant data to the DATADRYAD website. Consequently, the present study, a secondary analysis based on the above-mentioned database ^19^, further evaluated the association between height and risk of diabetes.

## Methods

### Data source

Data are available on the datadryad website (www.datadryad.org), which allow users to freely obtain original data. According to Dryad terms of service, the Dryad package was cited: Chen, Ying et al. (2018), Data from: association of body mass index (BMI) and age with incident diabetes in Chinese adults: a population-based cohort study, Dataset, https://doi.org/10.5061/dryad.ft8750v. In this database, continuous variables included age, height, weight, systolic blood pressure (SBP), diastolic blood pressure (DBP), alanine aminotransferase (ALT), aspartate transaminase (AST), fasting plasma glucose (FPG), total cholesterol (TC), triglyceride (TG), low-density lipoprotein cholesterol (LDL-C), high-density lipoprotein cholesterol (HDL-C) at baseline and follow-up time. Categorical variables consisted of gender, smoking status (current, past, never or unknown), drinking status (current, past, never or unknown), and family history of diabetes. The endpoint of interest was diabetes occurred during follow-up. In the initial study^19^, the copyright and ownership of the database have been waived. Consequently, this database can be employed to re-analysis without infringing the author's rights.

### Study population

**Figure 1:**
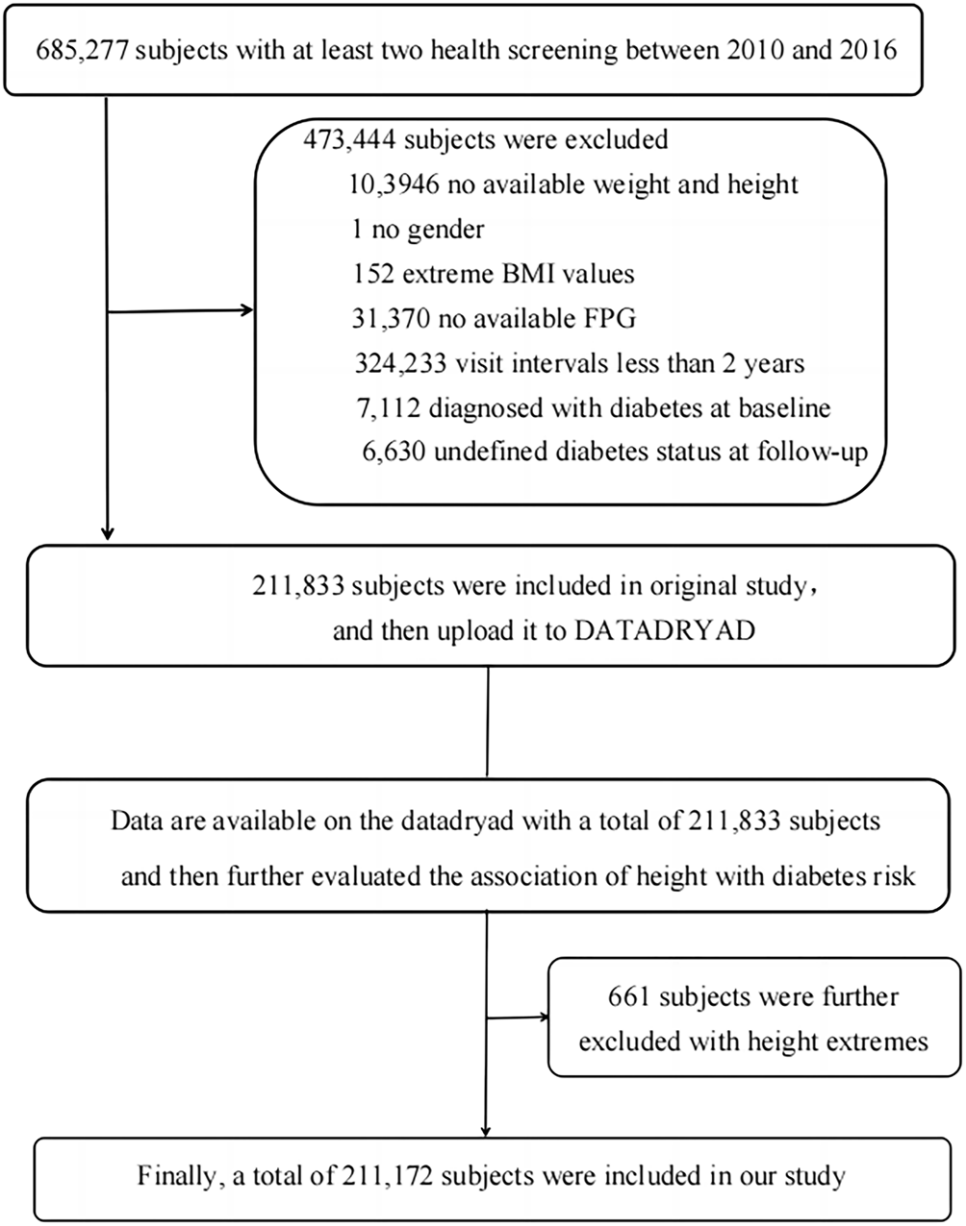
Flowchart depicting the screening of the study participants.

The initial study was conducted by Chen et al.^19^ Here, the study protocol was only briefly introduced, and the complete details were described in the original study^19^. This was a health screening project database established by Rich Healthcare Group, which contained 685,277 subjects who received at least two physical examinations from 2010 to 2016 across 32 locations in 11 cities of China. In the original study^19^, they had removed individuals who fulfilled any of the following exclusion criteria: missing data on FPG, height, or weight; BMI > 55 kg/m^2^ or < 15 kg/m^2^; follow-up period less than two years; a history of diabetes or the status of diabetes undetermined by the deadline. Finally, 211,833 subjects were identified in that study^19^. In the present study, to further elucidate the association of height and diabetes risk, we further excluded some height extremes (< mean—3 standard deviation (SD) or > mean + 3SD)^20^. Finally, a total of 211,172 participants were yielded in the analysis. A flowchart of the screening of the study participants was shown in Fig. 1.

### Variables definition and assessment

A standardized questionnaire was employed to gather information on anthropometric data, disease history, smoking status, and alcohol intake. Height and weight were measured by trained staff and were required to be accurate to 0.1 cm and 0.1 kg, respectively. BMI was defined as weight (kg) divided by height square (m^2^). Fasting state required fasting for at least 10 h. Blood biochemical indexes, such as serum lipids, FPG, ALT, and AST, were detected by automatic biochemical analyzer (Beckman 5800). The above data were obtained by trained professionals.

### Ascertainment of diabetes

The endpoint of interest was the first diagnosis of diabetes during follow-up, which was identified by FPG ≥ 7.0 mmol/L and/or self-reported diabetes. The determination of diabetes depended on the date of first diagnosis or last visit.

### Ethical approval

In the initial study^19^, the authors stated that the study was approved by the Rich Healthcare Group Review Board and abided by the declaration of Helsinki. Because the database for this study was publicly available, participant identities were anonymized, and information was retrieved retrospectively, as reported elsewhere^21,22^, informed consent was not required.

### Statistical analysis

Categorical variables were expressed as numbers (percentage) and compared with Chi square test, while continuous variables were presented with mean ± SD or median (25–75 percentile) and compared with one-way ANOVA or Kruskal Wallis H test, respectively. The independent effect of height on diabetes risk was evaluated by cox proportional hazard model. Considering the physiological differences of height between men and women, cox hazard regression analyses were performed in gender stratification to determine gender-specific associations. Three models were employed to evaluate the relationship of height and diabetes risk adjusting for potential confounding factors including age, weight, metabolic risk factors (FPG, LDL-C, HDL-C, TG, TC, AST, ALT, SBP, and DBP), lifestyle risk factors (drinking and smoking status) and genetic risk factors (family history of diabetes). The specific adjustment strategies were as follows: model 1 was adjusted for none; model 2 was adjusted for age, weight, family history of diabetes, smoking, and drinking; model 3 was further adjusted for FPG, LDL-C, HDL-C, TC, TG, ALT, AST, SBP and DBP on the basis of model 2. For the missing data of covariates, the missing data for categorical variable was treated as an independent group, and the corresponding mean or median was used to supplement for continuous variable^23^. Furthermore, a generalized additive model was implemented to explore the nonlinear relationship of height and diabetes risk. To evaluate the impact of other variables, subgroup analyses were conducted based on stratification by age, BMI, family history of diabetes, smoking status, drinking status, DBP, SBP, and FPG.

All statistical analyses were conducted using statistical software packages R (http://www.r-project.org, The R Foundation) and EmpowerStats (http://www.empowerstats.com, X&Y Solutions, Inc., Boston, MA) with a 2-sided significance threshold of *P*-value < 0.05.

## Results

### Baseline characteristics

Among the 211,833 participants, 357 males and 304 females with height extremes were eliminated, and the remaining 211,172 subjects (54.82% male) were yielded for the final data analysis. The mean age of men and women was 41.80 ± 12.28 years and 42.32 ± 12.90 years, respectively. The average height of men and women was 160.09 ± 5.57 cm and 171.66 ± 6.17 cm, respectively. During an average 3.12-year follow-up, 4156 (1.97%) subjects developed diabetes. As shown in Table 1, the baseline characteristics of population were demonstrated according to quintiles of height. Compared to short individuals, taller individuals, both male and female, were generally younger, had slightly lower blood pressure and FPG levels.

**Table 1:** Baseline characteristics of population according to quintiles of height.

Characteristics	Men (n = 115,766)					Women (n = 95,406)				
	Quintiles of height (cm)					Quintiles of height (cm)				
	153.0 − < 166.4	166.4 − < 169.9	169.9 − < 172.9	172.9 − < 176.9	176.9 − 190.4	143.1 − < 155.4	155.4 − < 158.4	158.4 − < 161.4	161.4 − < 164.9	164.6 − 177.0
N	22,760	19,773	23,711	25,511	24,011	18,899	18,200	19,852	18,542	19,913
Age, year	47.45 ± 14.86	44.03 ± 13.17	42.36 ± 12.45	40.48 ± 11.57	37.96 ± 10.22	46.75 ± 14.51	42.84 ± 12.49	41.38 ± 11.61	40.05 ± 10.86	38.20 ± 9.76
Weight, kg	64.77 ± 8.56	68.44 ± 8.91	70.74 ± 9.46	73.38 ± 10.06	78.36 ± 11.43	53.06 ± 7.47	55.03 ± 7.59	56.26 ± 7.71	57.83 ± 7.94	60.50 ± 8.41
BMI, kg/m^2^	24.38 ± 3.13	24.22 ± 3.15	24.16 ± 3.22	24.08 ± 3.29	24.10 ± 3.42	22.86 ± 3.21	22.34 ± 3.08	22.00 ± 3.01	21.78 ± 2.99	21.47 ± 2.92
SBP, mmHg	124.90 ± 16.87	122.98 ± 15.81	122.64 ± 15.29	122.00 ± 14.87	121.84 ± 14.35	118.44 ± 18.80	114.90 ± 16.61	113.87 ± 15.80	112.97 ± 14.97	112.22 ± 14.07
DBP, mmHg	77.39 ± 10.94	76.78 ± 10.71	76.77 ± 10.63	76.42 ± 10.50	75.93 ± 10.30	72.27 ± 10.93	71.35 ± 10.47	70.99 ± 10.12	70.68 ± 9.87	70.66 ± 9.85
FPG, mmol/L	5.06 ± 0.64	5.00 ± 0.63	4.97 ± 0.63	4.95 ± 0.62	4.91 ± 0.61	4.92 ± 0.60	4.87 ± 0.59	4.83 ± 0.58	4.81 ± 0.57	4.79 ± 0.57
TC, mmol/L	4.78 ± 0.89	4.75 ± 0.88	4.74 ± 0.88	4.70 ± 0.87	4.67 ± 0.87	4.82 ± 0.94	4.70 ± 0.90	4.67 ± 0.89	4.63 ± 0.88	4.60 ± 0.86
TG, mmol/L	1.31 (0.90–1.89)	1.33 (0.90–1.92)	1.30 (0.90–1.89)	1.30 (0.90–1.88)	1.29 (0.89–1.84)	0.99 (0.70–1.40)	0.90 (0.65–1.34)	0.88 (0.64–1.27)	0.85 (0.62–1.23)	0.82 (0.61–1.17)
HDL-C, mmol/L	1.33 ± 0.21	1.33 ± 0.21	1.33 ± 0.21	1.32 ± 0.20	1.32 ± 0.20	1.42 ± 0.23	1.42 ± 0.23	1.43 ± 0.24	1.43 ± 0.23	1.43 ± 0.25
LDL-C, mmol/L	2.79 ± 0.50	2.79 ± 0.50	2.79 ± 0.50	2.78 ± 0.49	2.77 ± 0.49	2.81 ± 0.54	2.77 ± 0.52	2.75 ± 0.52	2.73 ± 0.51	2.72 ± 0.50
ALT, U/L	22.30 (16.30–33.00)	23.00 (16.90–34.00)	23.00 (16.70–34.00)	23.00 (16.40–34.00)	23.10 (16.70–35.30)	15.00 (11.40–20.00)	14.00 (11.00–19.10)	13.80 (10.90–18.60)	13.50 (10.70–18.10)	13.00 (10.30–17.60)
AST, U/L	23.98 ± 7.53	23.68 ± 6.86	23.69 ± 7.38	23.46 ± 7.27	23.50 ± 8.16	22.41 ± 5.58	21.99 ± 5.32	21.83 ± 5.72	21.61 ± 5.32	21.39 ± 5.40
Smoker, %										
Current	2523 (11.09%)	2206 (11.15%)	2515 (10.61%)	2515 (9.86%)	2256 (9.40%)	2 (0.01%)	7 (0.04%)	9 (0.05%)	5 (0.03%)	4 (0.02%)
Past	487 (2.14%)	444 (2.25%)	506 (2.13%)	546 (2.14%)	542 (2.26%)	4 (0.02%)	5 (0.03%)	3 (0.02%)	6 (0.03%)	4 (0.02%)
Never	5278 (23.18%)	4307 (21.78%)	5029 (21.21%)	5074 (19.89%)	4830 (20.12%)	4424 (23.41%)	4319 (23.73%)	4310 (21.70%)	3936 (21.23%)	3943 (19.80%)
Unknown	14,472 (63.59%)	12,816 (64.82%)	15,661 (66.05%)	17,376 (68.11%)	16,383 (68.22%)	14,469 (76.56%)	13,869 (76.20%)	15,530 (78.23%)	14,595 (78.71%)	15,962 (80.16%)
Drinker, %										
Current	341 (1.50%)	255 (1.29%)	261 (1.10%)	244 (0.96%)	217 (0.90%)	5 (0.03%)	7 (0.04%)	4 (0.02%)	3 (0.02%)	10 (0.05%)
Past	1554 (6.83%)	1441 (7.29%)	1798 (7.58%)	1853 (7.26%)	1739 (7.25%)	98 (0.52%)	112 (0.62%)	117 (0.59%)	124 (0.67%)	99 (0.50%)
Never	6393 (28.09%)	5261 (26.60%)	5991 (25.27%)	6038 (23.67%)	5672 (23.62%)	4327 (22.90%)	4212 (23.14%)	4201 (21.16%)	3820 (20.60%)	3842 (19.29%)
Unknown	14,472 (63.58%)	12,816 (64.82%)	15,661 (66.05%)	17,376 (68.11%)	16,383 (68.23%)	14,469 (76.55%)	13,869 (76.20%)	15,530 (78.23%)	14,595 (78.71%)	15,962 (80.16%)
Family history of diabetes, %										
No	22,533 (99.00%)	19,515 (98.70%)	23,356 (98.50%)	25,131 (98.51%)	23,601 (98.29%)	18,411 (97.42%)	17,669 (97.08%)	19,291 (97.17%)	17,997 (97.06%)	19,337 (97.11%)
Yes	227 (1.00%)	258 (1.30%)	355 (1.50%)	380 (1.49%)	410 (1.71%)	488 (2.58%)	531 (2.92%)	561 (2.83%)	545 (2.94%)	576 (2.89%)

A*LT* Alanine aminotransferase, *AST* Aspartate transaminase, *BMI* Body mass index, *DBP* Diastolic blood pressure, *FPG* Fasting plasma glucose, *HDL-C* High-density lipoprotein cholesterol, *LDL-C* Low-density lipoprotein cholesterol, *SBP* Systolic blood pressure, *TC* Total cholesterol, *TG* Triglyceride.

Values are shown as mean ± SD, median (interquartile range) or n (percent).

### Univariate analysis

As shown in Supplementary Table S1, univariate analyses showed that these factors, including age, weight, BMI, SBP, DBP, FPG, LDL-C, TG, TC, AST, ALT, family history of diabetes, drinking, and smoking, were positively associated with diabetes risk. However, height and HDL-C were inversely correlated with future diabetes risk among men. In addition to the fact that the number of smokers and drinkers was too small for statistical analysis, similar results were observed among women.

### Association between height and diabetes risk

In multivariable-adjusted cox hazard regression models, as shown in Table 2, an inverse association of height with diabetes risk was observed among men and women [HR per 10 cm (95% CI), 0.78 (0.73–0.83) and 0.76 (0.68–0.86), respectively]. Sensitivity analysis was performed by treating height as a categorical variable (quintiles) to assess the robustness of the results. The overall trend was consistent in three models from the first quintile (Q1) to the fifth quintile (Q5). In fully adjusted model 3, taking Q1 as a reference, individuals with height in Q5 reduced the risk of diabetes by 34% (HR 0.66; 95% CI 0.58–0.75) and 35% (HR 0.65; 95% CI 0.53–0.81) in men and women respectively.

**Table 2:** Cox hazard regression results of association between height and diabetes risk.

Quintiles of height	No. of participants N (%)	No. of events N (%)	Model 1 HR (95% CI)	Model 2 HR (95% CI)	Model 3 HR (95% CI)
Male					
Q1	22,760 (19.7%)	789 (3.4%)	*Ref*	*Ref*	*Ref*
Q2	19,773 (17.1%)	560 (2.8%)	0.78 (0.70, 0.87)	0.75 (0.67, 0.84)	0.83 (0.74, 0.92)
Q3	23,711 (20.5%)	590 (2.5%)	0.68 (0.61, 0.75)	0.62 (0.55, 0.69)	0.75 (0.67, 0.84)
Q4	25,511 (22.0%)	564 (2.2%)	0.60 (0.54, 0.67)	0.51 (0.45, 0.57)	0.72 (0.64, 0.81)
Q5	24,011 (20.7%)	489 (2.0%)	0.56 (0.50, 0.63)	0.38 (0.34, 0.44)	0.66 (0.58, 0.75)
*P* for trend			< 0.0001	< 0.0001	< 0.0001
Continuous HR per 10 cm			0.71 (0.67, 0.75)	0.56 (0.52, 0.60)	0.78 (0.73, 0.83)
Female					
Q1	18,899 (19.8%)	390 (2.1%)	*Ref*	*Ref*	*Ref*
Q2	18,200 (19.1%)	244 (1.3%)	0.62 (0.53, 0.73)	0.72 (0.61, 0.85)	0.77 (0.65, 0.90)
Q3	19,852 (20.8%)	214 (1.1%)	0.49 (0.42, 0.58)	0.64 (0.53, 0.76)	0.79 (0.67, 0.94)
Q4	18,542 (19.4%)	175 (0.9%)	0.43 (0.36, 0.52)	0.55 (0.46, 0.67)	0.78 (0.65, 0.95)
Q5	19,913 (20.9%)	141 (0.7%)	0.32 (0.26, 0.39)	0.42 (0.34, 0.52)	0.65 (0.53, 0.81)
*P* for trend			< 0.0001	< 0.0001	0.0002
Continuous HR per 10 cm			0.47 (0.42, 0.52)	0.58 (0.52, 0.66)	0.76 (0.68, 0.86)

Model 1 adjusted for none; Model 2 adjusted for age, weight, smoking, drinking and family history of diabetes; Model 3 adjusted for age, weight, smoking, drinking, family history of diabetes, fasting blood glucose, low-density lipoprotein cholesterol, total cholesterol, triglyceride, high-density lipoprotein cholesterol, alanine aminotransferase, aspartate transaminase, systolic blood pressure and diastolic blood pressure.

*CI* confidence interval, *HR* hazard ratio, *Q* Quintile, *Ref* Reference.

### Nonlinear relationship of height with diabetes risk

**Figure 2:**
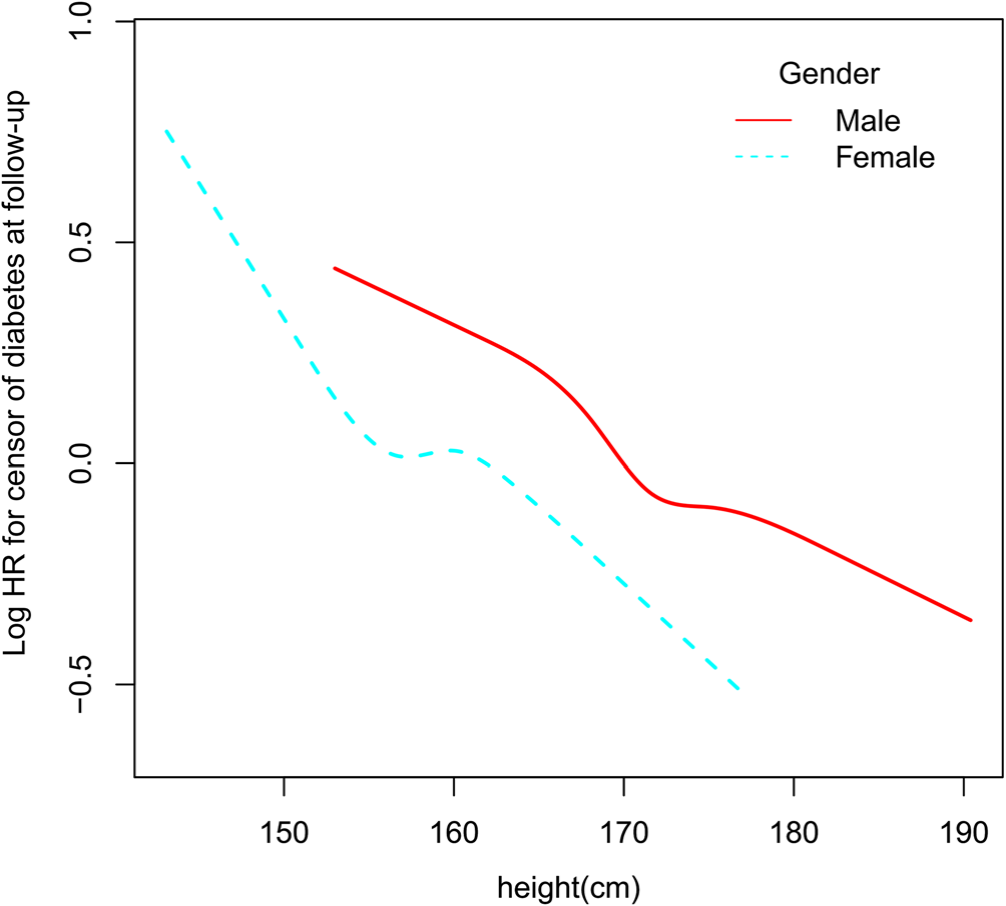
An approximate linear relationship of height with future diabetes risk stratified by sex. *Note*: the model was adjusted for age, weight, smoking, drinking, family history of diabetes, fasting blood glucose, total cholesterol, low-density lipoprotein cholesterol, triglyceride, alanine aminotransferase, aspartate transaminase, systolic blood pressure and diastolic blood pressure.

Considering the gender differences in height, nonlinear relationship of height with diabetes risk was explored according to gender (Fig. 2). After adjusting for potential confounding factors, an approximate linear relationship was observed both in men and women, which could be clearly observed that a significant inverse association was detected in both men and women.

### Subgroup analysis

In the prespecified subgroup analysis, especially the variables age, BMI, and FPG, as shown in Supplementary Tables 2–4, were significantly positively associated with diabetes risk, indicating that they are important risk factors for diabetes onset. As shown in Table 3, most stratification showed an inverse association between height and diabetes risk. The results of subgroup analyses showed that height demonstrated a inverse association with future diabetes risk except for those age < 30 or age ≥ 70. It is important to note that the results in the subgroup of subjects younger than 30 years may be unstable, with too many confounders adjusted and too few outcome events (52 in men and 23 in women). The inverse relationship was only significant in non-obese men but not in obese men, whereas, it was evident in women regardless of obesity status. The stronger inverse association was observed in men and women with FPG < 6.1 mmol/L, whereas, no significant associations were detected in subjects with FPG ≥ 6.1 mmol/L. Additional stratified analyses including SBP, DBP, smoking status, drinking status, and family history of diabetes were presented, and the majority of stratification demonstrated the inverse association of height with diabetes risk.

**Table 3:** Subgroup analyses of associations of height and risk of diabetes.

Stratified variables	Men (n = 115,766)			Women (n = 95,406)		
	No	No. of events	HR (95% CI)	No	No. of events	Model 3 HR (95% CI)
Age (year)						
≥ 20, < 30	15,767	52	0.92 (0.53, 1.62)*	12,755	23	0.89 (0.43, 1.71)*
≥ 30, < 40	44,911	430	0.75 (0.63, 0.89)	37,824	114	0.54 (0.37, 0.84)
≥ 40, < 50	23,696	613	0.66 (0.57, 0.77)	21,641	176	0.58 (0.44, 0.77)
≥ 50, < 60	17,423	939	0.82 (0.72, 0.93)	12,522	282	0.78 (0.62, 0.98)
≥ 60, < 70	9641	630	0.82 (0.73, 0.95)	7837	338	0.75 (0.60, 0.93)
≥ 70	4328	328	0.90 (0.73, 1.12)	2827	231	1.09 (0.82, 1.44)
BMI (kg/m^2^)						
< 24	55,993	649	0.75 (0.61, 0.92)	72,515	448	0.70 (0.58, 0.84)
≥ 24, < 28	46,035	1445	0.66 (0.56, 0.78)	18,548	481	0.79 (0.65, 0.96)
≥ 28	13,738	898	0.97 (0.83, 1.13)	4343	235	0.79 (0.59 1.00)
FPG (mmol/L)						
< 6.1	110,690	1556	0.61 (0.56, 0.68)	93,322	2084	0.64 (0.55, 0.75)
≧ 6.1	5076	1436	0.93 (0.84, 1.03)	673	491	0.84 (0.70, 1.02)
SBP (mmHg)						
< 140	101,290	2139	0.78 (0.72, 0.85)	88,594	762	0.73 (0.63, 0.85)
≥ 140	14,476	853	0.80 (0.70, 0.91)	6812	402	0.82 (0.66, 0.99)
DBP (mmHg)						
< 90	103,308	2324	0.77 (0.71, 0.83)	90,872	993	0.76 (0.67, 0.87)
≥ 90	12,458	668	0.83 (0.72, 0.96)	4534	171	0.83 (0.58, 1.17)
Smoker						
Now	4568	137	0.86 (0.73, 1.00)	27	1	__§
Once	912	22	0.57 (0.39, 0.83)	22	2	__§
Never	25,411	663	0.76 (0.65, 0.88)	20,684	248	__§
Not recorded	84,875	2170	0.77 (0.72, 0.84)	73,512	912	
Drinker						
Now	567	13	0.54 (0.30, 0.97)	29	0	__§
Once	3312	89	0.74 (0.55, 1.00)	550	5	__§
Never	27,012	720	0.79 (0.68, 0.91)	20,402	246	__§
Not recorded	84,875	2170	0.78 (0.72, 0.85)	74,425	913	__§
Family history of diabetes						
No	114,136	2891	0.79 (0.73, 0.84)	92,705	1095	0.76 (0.67, 0.86)
Yes	1630	101	0.63 (0.41, 0.96)	2701	69	0.71 (0.43, 1.18)

Hazard ratios for diabetes were comparison of incident diabetes per 10 cm difference in height. The models were adjusted for age, weight, smoking, drinking, family history of diabetes, fasting blood glucose, low-density lipoprotein cholesterol, total cholesterol, triglyceride, alanine aminotransferase, aspartate transaminase, systolic blood pressure and diastolic blood pressure except the corresponding stratification variable.

^§^The model failed because of the small sample size.

*The results in the subgroup of subjects younger than 30 years may be unstable, with too many confounders adjusted and too few outcome events.

*BMI* body mass index, *FPG* fasting plasma glucose, *SBP s*ystolic blood pressure, *DBP* diastolic blood pressure, *HR* hazard ratio, *CI* confidence interval.

### Sensitivity analysis

Considering that height may decrease slightly with age, we further removed participants ≥ 70 years old (n = 7155) for sensitivity analysis. As shown in Supplementary Table 5, the association between height and diabetes risk remained stable. In multivariable-adjusted cox hazard regression model, an inverse association of height with diabetes risk was observed among men and women [HR per 10 cm (95% CI), 0.78 (0.73–0.84) and 0.73 (0.64–0.84), respectively].

## Discussion

In this large-scale cohort study based on a Chinese population, we observed that higher height was related to reduced risk of diabetes in both women and men, even after adjusting for potential confounding factors. Compared with the lowest quintile, participants with height in the top quintile showed a 34% and 35% lower risk of diabetes in men and women, respectively. Additionally, similar findings were found across the majority of subgroup analyses. The inverse association was observed only in the subgroup with normal FPG, but disappeared in that with impaired fasting glucose (IFG) both in men and women. The inverse relationship was only significant in non-obese men, whereas, it was evident in women regardless of obesity status. Moreover, the inverse correlation was more pronounced in participants with age < 70 years.

Although some epidemiological studies have reported the relationship of height with diabetes risk, the results are inconsistent across different races and populations. A meta-analysis of five cohort studies and four cross-sectional studies, published in 2012, showed that height was negatively correlated with T2DM only in women (RR = 0.83; 95% CI 0.73–0.95), but not in men (RR = 0.87; 95% CI 0.71–1.07)^18^. However, heterogeneity was high among these included studies. Likewise, in a recent cross-sectional study of 3,241 Namibians^17^, similar results were found that the inverse correlation was observed only in women (OR = 0.96; 95% CI 0.94–0.99) rather than in men (OR = 1.02; 95% CI 0.98–1.05). However, in an Israeli cohort study of 32,055 non-diabetic young men with follow-up of 6.3 years^24^, subjects with height below the 10th percentile showed a 64% increased risk of diabetes compared with the 75th percentile of height (HR = 1.64; 95% CI 1.09–2.46). Moreover, consistent with our results, a nationwide population-based cohort study^15^, which was conducted in 21,122,422 South Koreans followed up for 5.6 years, showed that, compared with the top quintile group, subjects in the lowest quintile of height increased the risk of diabetes by 23% (HR = 1.23; 95% CI 1.22–1.24), and similar results were obtained in analysis by sex. Similarly, in a European cohort of 2029 non-diabetic individuals with 7-year follow-up^13^, each 10 cm increase in adult height was correlated with a 41% and 33% reduction of diabetes risk among men and women, respectively. Likewise, in this present study, a 10-cm increase in height was related to 22% and 24% reduced odds of developing diabetes in men and women, respectively. Besides, compared with the lowest quintile of height, the risk of diabetes in the highest quintile of height decreased by 34% and 35% in men and women, respectively. Our results are meaningful because we provide evidence for the Chinese population.

Notably, a similar paper published by Song et al., using the same dataset with us to examine height and diabetes risk^25^. However, despite the same database we used, the conclusions drawn are not entirely consistent. A gender difference was found in the article by Song et al., and the inverse relationship was found only in women rather than in men. There were the possible reasons leading to our different conclusions. Firstly, the sample sizes were different. In our study, to further elucidate the association of height and diabetes risk, we further excluded some height extremes (< mean—3 standard deviation (SD) or > mean + 3SD). Finally, a total of 211,172 participants were yielded in the analysis. While, in the study by Song et al., they further excluded 95,172 (44.93%) participants with loss of baseline lipid parameters, and finally only 116,661 participants were included. Lipid parameters as confounding factors, missing data could be imputed by modern statistical methods, and crude direct deletion would lead to selection bias. The flow chart and the comparison of data inclusion were detailed in Supplementary Fig. 1. Secondly, the confounders adjusted for were not identical. Since height was a component of BMI, directly adjusting for BMI would attenuate the effect of height on the risk of diabetes. The confounding factor adjusted in our study was body weight rather than BMI. Thirdly, another large Asian cohort study^15^ involving 21,122,422 Koreans showed similar results to ours rather than a gender difference as concluded by Song et al. Finally, we used modern statistical methods to conduct detailed subgroup analysis and explore the nonlinear relationship to repeatedly demonstrate the reliability of the core results.

The inverse relationship was significant only in individuals with FPG < 6.1 mmol/L rather than FPG ≥ 6.1 mmol/L, as shown in Table 3, which was consistent with a Korean population study^15^. The possible explanation is that, for individuals already in prediabetes, the effects of IFG overwhelms the effect of short stature, indicating that IFG exerts a greater impact on diabetes than height. In fact, in individuals with prediabetes, both islet β cell dysfunction and insulin resistance are already present, thus greatly increasing the risk of developing type 2 diabetes later in life^26^. A meta-analysis of 11 studies involving 3837 subjects with IFG showed a cumulative 5-year incidence of type 2 diabetes of 26%, and this rate was higher in the Chinese population, fluctuating from 25 to 38%^27^. In another study with a mean follow-up period of 6.4 years, the cumulative incidence of diabetes was 64.5% in patients with prediabetes and 4.5% in those with baseline normal glucose levels^28^. Thus, the effect of height on diabetes risk in participants with IFG was masked by the overwhelming risk factor of IFG. Similarly, the inverse relationship was only significant in non-obese men, but not in obese men (HR = 0.97, 95% CI 0.83–1.13), whereas, it was evident in women regardless of obesity status. One likely interpretation for this finding is that increased body fat and subsequent early puberty in girls may accelerate bone maturity and affect the final height^29^. However, in boys, childhood obesity may be related to delayed puberty and higher height^30,31^. Furthermore, the inverse association was not observed in subjects with age > 70 years. A conceivable explanation is that the elderly usually face more complex competitive risk factors for diabetes than the young, which may attenuate the proportional impact of height on diabetes risk. In addition, considering that height may decrease slightly with age, sensitivity analyses excluding participants with age ≥ 70 showed that the inverse relationship remained stable in both men and women.

The increasing evidence has indicated the inverse association of height with diabetes risk, yet, the mechanism remains unclear. Animal experiments have found that the structure and function of tissues and organs involved in glucose metabolism have changed during organ formation or early life after nutritional restriction^7^. Other factors, including low birth weight (including premature birth or intrauterine growth restriction)^32,33^, childhood nutritional status^34,35^, and growth-related hormones [estrogen and insulin-like growth factor-I (IGF-1)], are thought to be potential ways to link short stature with the risk of diabetes in adulthood. Sex-specific differences in height and diabetes risk may be mediated by the BMI, particularly rapid weight gain before puberty. Rapid weight gain in girls before puberty may lead to earlier menarche age, accelerate bone maturation, and ultimately affect the final attained height^29^. However, in boys, childhood obesity may be related to delayed puberty and heightened height^30,31^. In addition, studies have shown that short individuals have more pronounced insulin resistance compared with tall individuals^36–38^. Higher adult height was also associated with higher IGF-I concentrations^39^, which could increase insulin sensitivity^40^. In short, height is a comprehensive assessment of nutrition and environment in utero and childhood, and affects the development of diabetes. However, the in-depth mechanism between height and diabetes risk needs further to be elucidated.

Several limitations should be noteworthy. First, newly diagnosed diabetes was determined by FPG ≥ 7.0 mmol/L and/or self-reported diabetes. Consequently, those who met the diagnostic criteria for postprandial diabetes may be missing. Besides, there was no distinction between the types of diabetes. Second, the average follow-up time of 3.12 years was a relatively short time to observe the relationship of height with diabetes development. Third, even though some potential confounding factors have been adjusted for, confounding due to unmeasured differences in socio-economic or other factors may still exist. Fourth, adult height was used as a comprehensive evaluation indicator for children's nutritional status instead of direct parameters of early childhood malnutrition, which inevitably led to misclassification of exposure status. On the one hand, an individual who has experienced childhood stunted growth and subsequent catch-up growth was indistinguishable from a well-nourished person with normal growth. On the other hand, shorter adult height may be related to other factors independent of early nutritional status, such as endocrine, metabolic and genetic factors^41^. Nevertheless, adult height has proved to be a measure of accumulated net nutrition at the population level^42^. Children with stunted growth, after all, have only a small chance for catch-up with growth^3,10^. Finally, this study was conducted among Chinese adults over 20 years old. Thus, it should be cautious to generalize these findings to other age or ethnicity groups.

In summary, relying on this large cohort study, we observed that height was inversely associated with the risk of diabetes in Chinese adults. Besides, this association appears to be more pronounced in individuals with FPG < 6.1 mmol/L, aged < 70 years, and men with BMI < 28 kg/m^2^. However, further studies are required to determine whether this association is causal and to better understand its underlying mechanisms.

### Supplementary Information

Supplementary Information.

## Data Availability

Data can be downloaded from ‘DATADRYAD’ database (www.Datadryad.org).
