# Supplementary material for: Inverse association between adult height and diabetes risk in a cohort study of Chinese population
Source: Sci Rep. 2023 Nov 27;13:20835. doi: 10.1038/s41598-023-47474-1 (PMC10681985; doi:10.1038/s41598-023-47474-1)
Supplement: Supplementary file 1 — Supplementary Information. [file 41598_2023_47474_MOESM1_ESM.pdf]

Inverse association between adult height and Diabetes risk in a cohort study of Chinese population

Xiaoli Li[1], Tiantian Cheng[2], Lina Leng[1], Guangyao Song[2], Huijuan Ma[3]*

[1]Department of Rheumatology, Xingtai people's Hospital, Xingtai 054000, China

[2]Department of Endocrinology, Hebei General Hospital, Shijiazhuang 050051, China

[3]Department of Endocrinology, The First Hospital of Hebei Medical University, Shijiazhuang 050030, China

***Corresponding author: Huijuan Ma, Email: huijuanma76@163.com**

Supplementary Table S1. Univariate Cox regression analysis of association between potential risk factors and diabetes risk

Variables	Male		Female
	HR (95% CI)		HR (95% CI)
**Age, year**	1.06 (1.05-1.06)		1.08 (1.08-1.09)
**Height, cm**	0.97 (0.96-0.97)		0.93 (0.92-0.94)
**Weight, kg**	1.05 (1.04-1.05)		1.08 (1.07-1.08)
**BMI, kg/m^2**	1.22 (1.21-1.23)		1.26 (1.24-1.27)
**SBP, mmHg**	1.03 (1.03-1.03)		1.05 (1.05-1.05)
**DBP, mmHg**	1.04 (1.03-1.04)		1.05 (1.05-1.06)
**FPG, mmol/L**	9.33 (8.85-9.83)		12.74 (11.74-13.83)
**TC, mmol/L**	1.31 (1.26-1.36)		1.75 (1.66-1.84)
**TG, mmol/L**	1.25 (1.24-1.27)		1.48 (1.45-1.51)
**HDL-C, mmol/L**	0.75 (0.63-0.89)		0.52 (0.40-0.68)
**LDL-C, mmol/L**	1.19 (1.11-1.28)		1.90 (1.75-2.07)
**ALT, U/L**	1.01 (1.01-1.01)		1.01 (1.01-1.02)
**AST, U/L**	1.02 (1.01-1.02)		1.02 (1.01-1.02)
**Smoker, %**			
**Never**	*Ref*		__§
**Current**	1.90 (1.66, 2.18)		__§
**Past**	1.45 (1.14, 1.86)		__§
**Unknown**	1.48 (1.33, 1.64)		__§
**Drinker, %**			
**Never**	*Ref*		__§
**Current**	1.71 (1.28, 2.29)		__§
**Past**	1.08 (0.89, 1.34)		__§
**Unknown**	1.20 (1.01, 1.38)		__§
**Family history of diabetes, %**			
**No**	*Ref*		*Ref*
**Yes**	1.84 (1.51-2.25)		1.97 (1.54-2.51)

Note 1: §The model failed because of the small sample size.

Note 2: Cox hazard regression analyses were used to assess the association of potential risk factors with diabetes risk, and no additional confounders were adjusted for.

Abbreviation: ALT alanine aminotransferase, *AST* aspartate transaminase, *BMI* body mass index, *CI* confidence interval, *DBP* diastolic blood pressure, *FPG* fasting plasma glucose, *HDL-C* high-density lipoprotein cholesterol, *HR* hazard ratio, *LDL-C* low-density lipoprotein cholesterol, *Ref* reference, *SBP* systolic blood pressure, *TC* total cholesterol, *TG* triglyceride.

Supplementary Table S2. Cox hazard regression results of association between age and diabetes risk among participants.

Exposure	Men (n= 115,766)				Women (n=95,406)		
	Model 1<br>HR (95% CI)	Model 2<br>HR (95% CI)	Model 3<br>HR (95% CI)		Model 1<br>HR (95% CI)	Model 2<br>HR (95% CI)	Model 3<br>HR (95% CI)
Age (y), Continuous	1.06 (1.05, 1.06)	1.06 (1.05, 1.06)	1.03 (1.03, 1.04)		1.08 (1.08, 1.09)	1.07 (1.07, 1.08)	1.04 (1.03, 1.04)
Age (y), Categorical							
<30	*Ref*	*Ref*	*Ref*		*Ref*	*Ref*	*Ref*
≥30, <40	2.12 (1.59, 2.83)	1.78 (1.34, 2.38)	1.67 (1.25, 2.22)		1.19 (0.76, 1.86)	1.00 (0.64, 1.56)	0.92 (0.59, 1.44)
≥40, <50	5.49 (4.13, 7.28)	4.31 (3.24, 5.72)	2.94 (2.20, 3.91)		3.11 (2.01, 4.81)	2.21 (1.43, 3.41)	1.58 (1.02, 2.45)
≥50, <60	11.52 (8.71, 15.24)	9.12 (6.89, 12.05)	4.42 (3.33, 5.88)		9.30 (6.08, 14.23)	5.55 (3.62, 8.51)	2.42 (1.56, 3.73)
≥ 60, <70	14.92 (11.24, 19.80)	11.44 (8.62, 15.19)	4.50 (3.37, 6.01)		19.95 (13.08, 30.44)	10.79 (7.05, 16.52)	3.35 (2.16, 5.19)
≥70	18.97 (14.15, 25.41)	15.85 (11.83, 21.24)	6.34 (4.69, 8.56)		38.29 (24.94, 58.79)	19.27 (12.50, 29.71)	4.50 (2.86, 7.08)

Note: Model 1 adjusted for none; Model 2 adjusted for BMI, smoking, drinking and family history of diabetes; Model 3 adjusted for BMI, smoking, drinking, family history of diabetes, fasting blood glucose, low-density lipoprotein cholesterol, total cholesterol, triglyceride, high-density lipoprotein cholesterol, alanine aminotransferase, aspartate transaminase, systolic blood pressure and diastolic blood pressure.

Abbreviation: *CI* confidence interval, *HR* hazard ratio, *Ref* Reference

Supplementary Table S3. Cox hazard regression results of association between BMI and diabetes risk among participants.

Exposure	Men (n= 115,766)				Women (n=95,406)		
	**Model 1** HR (95% CI)	**Model 2** HR (95% CI)	**Model 3** HR (95% CI)		**Model 1** HR (95% CI)	**Model 2** HR (95% CI)	**Model 3** HR (95% CI)
BMI(kg/m^2), Continuous	1.22 (1.21, 1.23)	1.22 (1.21, 1.23)	1.10 (1.09, 1.12)		1.26 (1.24, 1.27)	1.19 (1.17, 1.21)	1.09 (1.08, 1.11)
BMI(kg/m^2), Categorical							
<24	*Ref*	*Ref*	*Ref*		*Ref*	*Ref*	*Ref*
≥24, <28	2.69 (2.45, 2.95)	2.25 (2.05, 2.47)	1.41 (1.28, 1.55)		4.46 (3.92, 5.07)	2.47 (2.16, 2.82)	1.64 (1.43, 1.88)
≥28	5.84 (5.28, 6.46)	5.18 (4.68, 5.73)	2.10 (1.88, 2.34)		9.79 (8.36, 11.46)	4.57 (3.88, 5.39)	2.03 (1.72, 2.41)

Note: Model 1 adjusted for none; Model 2 adjusted for age, smoking, drinking and family history of diabetes; Model 3 adjusted for age, smoking, drinking, family history of diabetes, fasting blood glucose, low-density lipoprotein cholesterol, total cholesterol, triglyceride, high-density lipoprotein cholesterol, alanine aminotransferase, aspartate transaminase, systolic blood pressure and diastolic blood pressure.

Abbreviation: *BMI* body mass index, *CI* confidence interval, *HR* hazard ratio, *Ref* Reference

Supplementary Table S4. Cox hazard regression results of association between FPG and diabetes risk among participants.

Exposure	Men (n= 115,766)				Women (n=95,406)		
	Model 1 HR (95% CI)	Model 2 HR (95% CI)	Model 3 HR (95% CI)		Model 1 HR (95% CI)	Model 2 HR (95% CI)	Model 3 HR (95% CI)
FPG (mmol/L), Continuous	9.33 (8.85, 9.83)	6.86 (6.48, 7.25)	6.51 (6.15, 6.89)		12.74 (11.74, 13.83)	7.61 (6.95, 8.33)	7.14 (6.51, 7.83)
FPG (mmol/L), Categorical							
<6.1	*Ref*	*Ref*	*Ref*		*Ref*	*Ref*	*Ref*
≥6.1	23.73 (22.08, 25.49)	12.69 (11.73, 13.74)	11.48 (10.60, 12.44)		42.45 (37.77, 47.70)	14.82 (13.02, 16.87)	13.00 (11.41, 14.82)

Note: Model 1 adjusted for none; Model 2 adjusted for age, body mass index, smoking, drinking and family history of diabetes; Model 3 adjusted for age, body mass index, smoking, drinking, family history of diabetes, low-density lipoprotein cholesterol, total cholesterol, triglyceride, high-density lipoprotein cholesterol, alanine aminotransferase, aspartate transaminase, systolic blood pressure and diastolic blood pressure.

Abbreviation: *FPG* fasting plasma glucose, *CI* confidence interval, *HR* hazard ratio, *Ref* Reference

Supplementary Table S5. Cox hazard regression results of association between height and diabetes risk among participants with age under 70 years

	**Model 1**	**Model 2**	**Model 3**
	**HR (95% CI)**	**HR (95% CI)**	**HR (95% CI)**
**Male**			
Continuous HR per 10cm	0.76 (0.71, 0.80)	0.54 (0.50, 0.58)	0.78 (0.73, 0.84)
Q1	*Ref*	*Ref*	*Ref*
Q2	0.73 (0.56, 0.95)	0.64 (0.49, 0.84)	0.80 (0.62, 1.05)
Q3	0.59 (0.46, 0.76)	0.47 (0.36, 0.60)	0.70 (0.55, 0.90)
Q4	0.50 (0.39, 0.64)	0.34 (0.27, 0.44)	0.60 (0.46, 0.77)
Q5	0.45 (0.35, 0.57)	0.24 (0.19, 0.31)	0.55 (0.43, 0.72)
P for trend	<0.0001	<0.0001	<0.0001
**Female**			
Continuous HR per 10cm	0.55 (0.49, 0.62)	0.51 (0.45, 0.58)	0.73 (0.64, 0.84)
Q1	*Ref*	*Ref*	*Ref*
Q2	0.72 (0.62, 0.83)	0.72 (0.62, 0.83)	0.93 (0.80, 1.08)
Q3	0.51 (0.42, 0.62)	0.51 (0.41, 0.62)	0.73 (0.60, 0.90)
Q4	0.48 (0.34, 0.68)	0.46 (0.32, 0.65)	0.79 (0.55, 1.13)
Q5	0.31 (0.10, 0.98)	0.21 (0.07, 0.67)	0.63 (0.20, 2.03)
P for trend	<0.0001	<0.0001	0.0042

Note: Model 1 adjusted for none; Model 2 adjusted for age, weight, smoking, drinking and family history of diabetes; Model 3 adjusted for age, weight, smoking, drinking, family history of diabetes, fasting blood glucose, low-density lipoprotein cholesterol, total cholesterol, triglyceride, high-density lipoprotein cholesterol, alanine aminotransferase, aspartate transaminase, systolic blood pressure and diastolic blood pressure.

Abbreviation: *CI* confidence interval, *HR* hazard ratio, *Q* Quintile, *Ref* Reference

Supplementary Fig 1

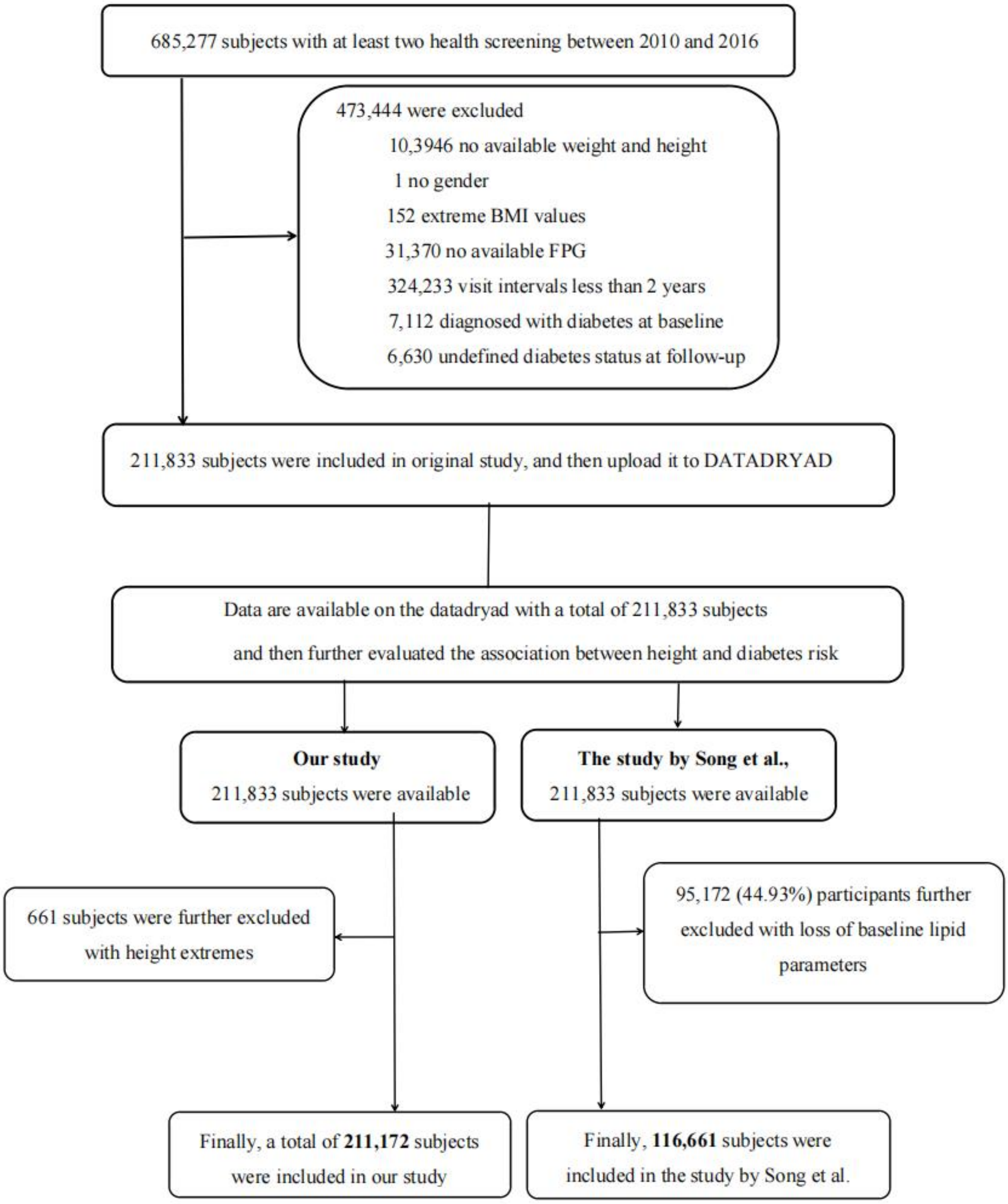

Supplementary Fig 1: The flow chart for the screening of study participants and the comparison with that of Song et al. Abbreviation: *BMI* body mass index, *FPG* fasting plasma glucose
